# RBiomirGS: an all-in-one miRNA gene set analysis solution featuring target mRNA mapping and expression profile integration

**DOI:** 10.7717/peerj.4262

**Published:** 2018-01-12

**Authors:** Jing Zhang, Kenneth B. Storey

**Affiliations:** 1 Schulich School of Medicine & Dentistry, University of Western Ontario, London, Canada; 2 Institute of Biochemistry, Departments of Biology and Chemistry, Carleton University, Ottawa, Canada

**Keywords:** Logistic regression, Pathway analysis, Transcriptome, Gene set enrichment, Molecular biology, Post-transcriptional regulation

## Abstract

**Background:**

With the continuous discovery of microRNA’s (miRNA) association with a wide range of biological and cellular processes, expression profile-based functional characterization of such post-transcriptional regulation is crucial for revealing its significance behind particular phenotypes. Profound advancement in bioinformatics has been made to enable in depth investigation of miRNA’s role in regulating cellular and molecular events, resulting in a huge quantity of software packages covering different aspects of miRNA functional analysis. Therefore, an all-in-one software solution is in demand for a comprehensive yet highly efficient workflow. Here we present RBiomirGS, an R package for a miRNA gene set (GS) analysis.

**Methods:**

The package utilizes multiple databases for target mRNA mapping, estimates miRNA effect on the target mRNAs through miRNA expression profile and conducts a logistic regression-based GS enrichment. Additionally, human ortholog Entrez ID conversion functionality is included for target mRNAs.

**Results:**

By incorporating all the core steps into one package, RBiomirGS eliminates the need for switching between different software packages. The modular structure of RBiomirGS enables various access points to the analysis, with which users can choose the most relevant functionalities for their workflow.

**Conclusions:**

With RBiomirGS, users are able to assess the functional significance of the miRNA expression profile under the corresponding experimental condition by minimal input and intervention. Accordingly, RBiomirGS encompasses an all-in-one solution for miRNA GS analysis. RBiomirGS is available on GitHub (http://github.com/jzhangc/RBiomirGS). More information including instruction and examples can be found on website (http://kenstoreylab.com/?page_id=2865).

## Introduction

MicroRNA (or miRNA) is a ∼22 nucleotide long small RNA species and is mostly recognized as a negative gene expression regulator on a post-transcriptional level (He & Hannon, 2004). miRNAs have been proposed as biomarkers and/or therapeutic targets for medical disorders such as drug-induced liver injury and cancer (Mitchell et al., 2008; Wang et al., 2009). Additionally, the primary structure of many miRNAs exhibits high level of conservation across species (Zhang & Storey, 2013), enabling smooth transfer of knowledge between different model systems.

Gene expression gene set (GS) analysis associates expression profiles with the functional outcome under specific experimental conditions and phenotypes. miRNA and coding gene expression GS analyses share the same general goal: to identify the significantly affected biological events from a given expression profile. The commonly used GS databases include gene ontology (GO) term (Ashburner et al., 2000) and KEGG (Kanehisa & Goto, 2000). Several GS techniques have been developed to directly incorporate differential expression (DE) results, such as gene set enrichment analysis (GSEA) (Subramanian et al., 2005). Even though it has been reported that these methods hold a more thorough and complete GS evaluation for coding genes (Mootha et al., 2003; Subramanian et al., 2005), the popular methods for miRNA research still rely on pre-selecting differentially expressed targets. Briefly, the commonly used miRNA GS analysis procedure starts with obtaining the list of the differentially expressed miRNAs, followed by searching for their target mRNAs, and then comparing the mRNA list with the GS databases (Long et al., 2013; Chen et al., 2013). However, it has been demonstrated that such method and its variations tend to exhibit bias of various origins (Khatri, Sirota & Butte, 2012; Bleazard et al., 2015). Moreover, the information on directionality from these methods is either indirect or lacking. One strategy to tackle the issue is to directly integrate miRNA DE results and transfer the information to the target mRNAs as a quantifiable metric.

There are a variety of computational analysis tools covering various aspects of miRNA studies, ranging from miRNA prediction, miRNA:mRNA interaction prediction and functional annotation (Gomes et al., 2013; Akhtar et al., 2016). As a result, multiple standalone tools are typically required to complete a miRNA GS workflow, e.g., mRNA target mapping, GS database preparation, GS enrichment, and results visualization. Practically, researchers usually face the challenge of constructing a pipeline for each project with multiple software packages and web services, which present incoherent connections between steps. Therefore, it is beneficial to establish a bioinformatic solution that searches multiple databases for mRNA target mapping and enables seamless navigation between analysis steps with minimal user intervention. Moreover, it is also critical to provide users with multiple entry points to the pipeline so that it is possible to customize and integrate only the functionalities necessary to their specific workflow. Here we present the R package RBiomirGS, a comprehensive miRNA GS analysis framework capable of performing the following tasks: (i) thorough target mRNA mapping, (ii) calculation of miRNA regulatory effect for target mRNAs, (iii) GS enrichment, and (iv) data visualization.

## Methods

**Figure 1:**
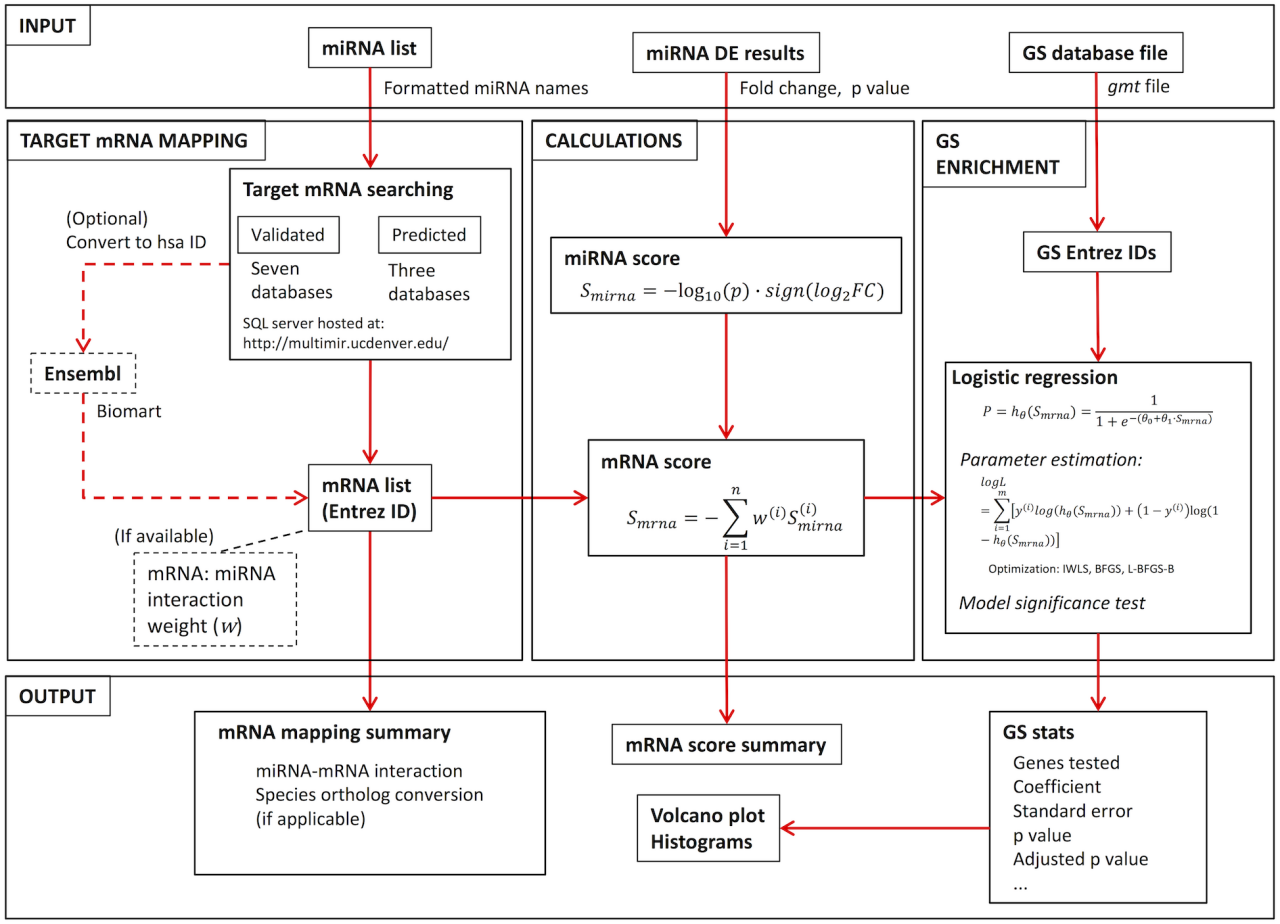
RBiomirGS workflow, showing input and output configuration, as well as all the functionality modules.

As shown in Fig. 1: users provide the miRNA identity list and associated DE results, as well as GS database file. The RNA mapping module takes the miRNA list and searches multiple databases for miRNA:mRNA interactions, resulting in either a validated or predicted target mRNA list. Fold change (FC) and *p* value from the miRNA DE list are then used to calculate a miRNA expression score for each miRNA measured, from which a miRNA impact score for target mRNAs is generated. With the mRNA score and GS database file, GS enrichment is then conducted using logistic regression. The package was built using R version 3.4.0 (R Core Team, 2017).

### Target mRNA mapping module

RBiomirGS features a target mRNA mapping module that utilizes multiple miRNA:mRNA interaction databases, whose information is hosted on a SQL server at University of Colorado Cancer Centre (http://multimir.ucdenver.edu/). Information for both predicted and validated miRNA:mRNA interactions can be retrieved from the server. Although a disease research-focused R interface was developed by the host institution for data query (Ru et al., 2014), we assembled our own module for a more general purpose miRNA:mRNA interaction search with additional code optimizations such as parallel computing. The current module takes advantage of multiple databases for a more complete mapping result. For the experimentally validated miRNA:mRNA interactions, miRecords, mirTarBase and TarBase were used (Xiao et al., 2009; Chou et al., 2016; Sethupathy, Corda & Hatzigeorgiou, 2006); whereas DIANA-microT-CDS, ElMMo, MicroCosm (http://www.ebi.ac.uk/enright-srv/microcosm/htdocs/targets/v5/info.html), miRanda (http://microrna.org), miRDB, PicTar, PITA, and TargetScan were searched for predicted interactions (Paraskevopoulou et al., 2013; Gaidatzis et al., 2007; Betel et al., 2008; Wang, 2008; Krek et al., 2005; Kertesz et al., 2007; Lewis, Burge & Bartel, 2005; Grimson et al., 2007; Friedman et al., 2009; Garcia et al., 2011). It is worth noting that DIANA-microT-CDS, PicTar, PITA and TargetScan are skipped for rat miRNAs. Currently, the mapping module supports human, rat and mouse miRNAs.

The core function of the target mRNA mapping module is *rbiomirGS_mrnascan*. The input file for this function is a list of miRNA names following the standard miRNA naming convention (http://www.mirbase.org/help/nomenclature.shtml). The function submits SQL queries to the server using the input miRNA list. The returned results are then output as both R list objects and as *csv* files to the working directory. By setting the species code (*hsa*, *rno* or *mmu* for human, rat or mouse, respectively), the function will search the databases accordingly. The argument *queryType* governs whether to search for validated or predicted interactions. For the output file, the universal column elements for both validated and predicted queries include Database, Mature miRNA miRBase accession number, Mature miRNA ID (name), Target gene symbol, Target gene Entrez ID, and Target gene Ensembl ID. The output results file will also contain column elements that are unique to the two query types.

### miRNA score and mRNA score

The core idea behind the current GS analysis strategy is to quantitatively estimate the miRNA regulatory effect on the target mRNAs, through which the miRNA impact on specific functional gene sets can be evaluated. Based on the initial study by Garcia-Garcia et al. (2016), a miRNA score is first calculated featuring the directionality presented in log FC (or log_2_FC), and log transformed *p* value (or −log_10_(*p*)). The equation is as follows:

(1) \begin{eqnarray*}{S}_{mirna}=-{\log \nolimits }_{10}p\cdot sign({\log \nolimits }_{2}FC)\end{eqnarray*}

As shown in Eq. (1), the *S*_*mirna*_ is a linear combination of the sign of log_2_FC and −log_10_(*p*). Integrating *p* value and the sign of log_2_FC ensures that both significance and directionality of the change are taken into consideration. *S*_*mirna*_ can be calculated either with or without prior filtering of miRNAs. Although either approaches are valid, using the whole miRNA list both reduces the influences from thresholding method and enables a GS analysis resembling the core principle of a competitive GS enrichment approach (De Leeuw et al., 2016), thereby ensuring high compatibility and statistical power.

Upon obtaining *S*_*mirna*_, the mRNA score (*S*_*mrna*_) can be calculated. The current calculation is a modification of the approach proposed by Garcia-Garcia et al. (2016). Such score is a quantitative representation of the potential regulatory effect on the target mRNAs from miRNAs. The equation is as follows:

(2) \begin{eqnarray*}{S}_{mrna}=-\sum _{i=1}^{n}{w}^{ \left( i \right) }{S}_{mirna}^{ \left( i \right) }\end{eqnarray*}

Equation (2) shows that the *S*_*mrna*_ of a mRNA is a sign reversed summation of the *S*_*mirna*_ of all the upstream miRNAs. The term *n* is the number of upstream miRNAs for the mRNA of interest; and *w* is the miRNA:mRNA affinity score, with values set as 1 by default, i.e., no difference between interactions. However, users can set such score using a numeric vector if available.

### Logistic regression-based GS enrichment

With *S*_*mrna*_ calculated with Eq. (2) and the GS database file, RBiomirGS uses logistic regression to enrich gene sets. Such approach is based on the core concept that a specific gene set is affected if its member genes are also regulated, either at the expression level or by influence from other regulatory factors such as miRNA. Practically, the goal is to assess if a gene can be categorized into a gene set solely based on its *S*_*mrna*_ value. As such, the enrichment algorithm models the probability of a gene with a specific *S*_*mrna*_ value belonging to a gene set. Mathematically, such probability is represented by the logistic regression sigmoid function (or hypothesis function):

(3) \begin{eqnarray*}P={h}_{\theta }({S}_{mrna})= \frac{1}{1+{e}^{-({\theta }_{0}+{\theta }_{1}\cdot {S}_{mrna})}} \end{eqnarray*}

As seen in Eq. (3), *P* is the aforementioned probability, which represents the hypothesis function of logistic regression with parameter vector *θ*. Transformation of Eq. (3) gives the equation below:

(4) \begin{eqnarray*}\log \nolimits \left( \frac{P}{1-P} \right) ={\theta }_{0}+{\theta }_{1}\cdot {S}_{mrna}\end{eqnarray*}

Equation (4) shows that the function is the log odds ratio of a gene belonging to the gene set of interest, given the associated *S*_*mrna*_ value. Coefficient *θ*_1_ stands for the change in the log odds ratio of the gene belonging to the gene set of interest by a unit change in *S*_*mrna*_.

The model parameter is estimated based on the principle of maximum likelihood (Fu & Li, 1993). Specifically, the following log likelihood function is maximized:

\begin{eqnarray*}\log \nolimits L=\sum _{i=1}^{m} \left[ {y}^{ \left( i \right) }\log \nolimits \left( {h}_{\theta }({S}_{mrna}) \right) + \left( 1-{y}^{ \left( i \right) } \right) \log \nolimits (1-{h}_{\theta }({S}_{mrna})) \right] \end{eqnarray*}

where *y* is the dummified membership to the gene set of interest for a gene, with 1 representing a member, 0 otherwise; *m* is the number of genes tested. RBiomirGS uses multiple optimization algorithms for finding the optimal parameter value for the model, including iteratively reweighted least square (IWLS), BFGS, and limited memory BFGS-B (L-BFGS-B) (Byrd et al., 1994; Roger, 1987; Wolke & Schwetlick, 1987). Such approach enables users to choose according to the volume of data and available computational power. RBiomirGS utilizes both generalized linear model (*glm*) function with logit link function natively included in R language, and a manual implementation of the logistic regression sigmoid function and log likelihood function. Specifically, the R native *glm* with logit link function uses IWLS by default; and the other two optimization methods work by applying general optimization function to the manual logistic regression implementation. To demonstrate the difference in performance with a specific dataset, an analysis of variance (ANOVA) test was conducted on the data from the case study using the statistical analysis R package RBioplot (Zhang & Storey, 2016).

The model significance test is carried out through a Wald test:

\begin{eqnarray*}z= \frac{\widehat{{\theta }_{1}}}{{s}_{\widehat{{\theta }_{1}}}} \end{eqnarray*}

where $\widehat{{\theta }_{1}}$ is the estimated model coefficient by maximum likelihood method; and ${s}_{\widehat{{\theta }_{1}}}$ represents the standard error for the estimated model coefficient. The GS *p* value is then obtained using the *z* score. For IWLS, *t* value is used instead to calculate the GS *p* value with one degree of freedom. All GS *p* values are then adjusted using a false discovery rate (FDR) (Benjamini & Hochberg, 1995).

The calculation of the scores and logistic regression analysis are achieved through the function *rbiomirgs_logistic*. The scores, along with the GS database file, are then passed to the logistic modelling process. Similar to the target mRNA mapping function, argument *objTitle* sets the file name prefix. The miRNA DE object can be set using the *mirna_DE* argument. The arguments *var_mirnaName*, *var_mirnaFC* and *var_mirnaP* are used to set the column elements for miRNA names, FC and *p* value, respectively. The target mRNA object can then be set using argument *mrnalist*. The *mrna_Weight* argument is used to incorporate the miRNA:mRNA interaction weight matrix, if available. The *gs_file* argument is used to set the GS database file. The parameter optimization algorithm can be set using argument *optim_method*. By default, FDR is used to adjust the GS *p* value via argument *p.adj*. The GS enrichment results are exported as a *csv* file. A *txt* file detailing iterations to convergence if either BFGS or L-BFGS-B is used. The function also outputs the result to the R environment so that data visualization can be carried out.

### Data visualization module

The current package includes a data visualization module utilizing the R package ggplot2 (Wickham, 2009). Specifically, the results can be plotted using bar graph and volcano plot. For bar graphs, two types of plots are featured in the package through function *rbiomirgs_bar*. Specifically, the horizontal bar graph inside the volcano plot depicts the overall distribution of the model coefficient (log odds ratio change per unit *S*_*mrna*_) for all the gene sets tested; whereas the vertical bar graph shows the gene sets with top model coefficient values. The function ranks the absolute coefficient values and plots the top user defined gene sets. The bar graph is model coefficient ± standard error. Users can choose to only plot the significantly enriched gene sets on the bar graphs, as shown in the case study. The volcano plot is carried out by the *rbiomirgs_volcano* function. Users can set the *p* value threshold and the number of top gene sets to display on the graph. Additionally, users can freely use other plotting packages to meet their specific data visualization needs.

## Results

We demonstrate the usage and performance of RBiomirGS using the liver data from a study assessing the role of miRNAs in facilitating daily torpor in hibernating South American marsupials (Hadj-Moussa et al., 2016). The original study assessed 85 miRNAs in the liver and skeletal muscle of aroused and torpid marsupials using a qPCR approach. Given that the miRNome has yet to be fully characterized for the marsupials, the study used mouse miRNA sequences for primer design. Such approach led to successful amplification of all 85 miRNAs in the marsupial. The case study used the mouse databases for target mRNA mapping. All output files can be downloaded and viewed from supplementary materials. The analysis was carried out on an Apple Macbook Pro computer with Intel Core i5 2.7 GHz dual-core CPU and 8 GB memory.

**Figure 2:**
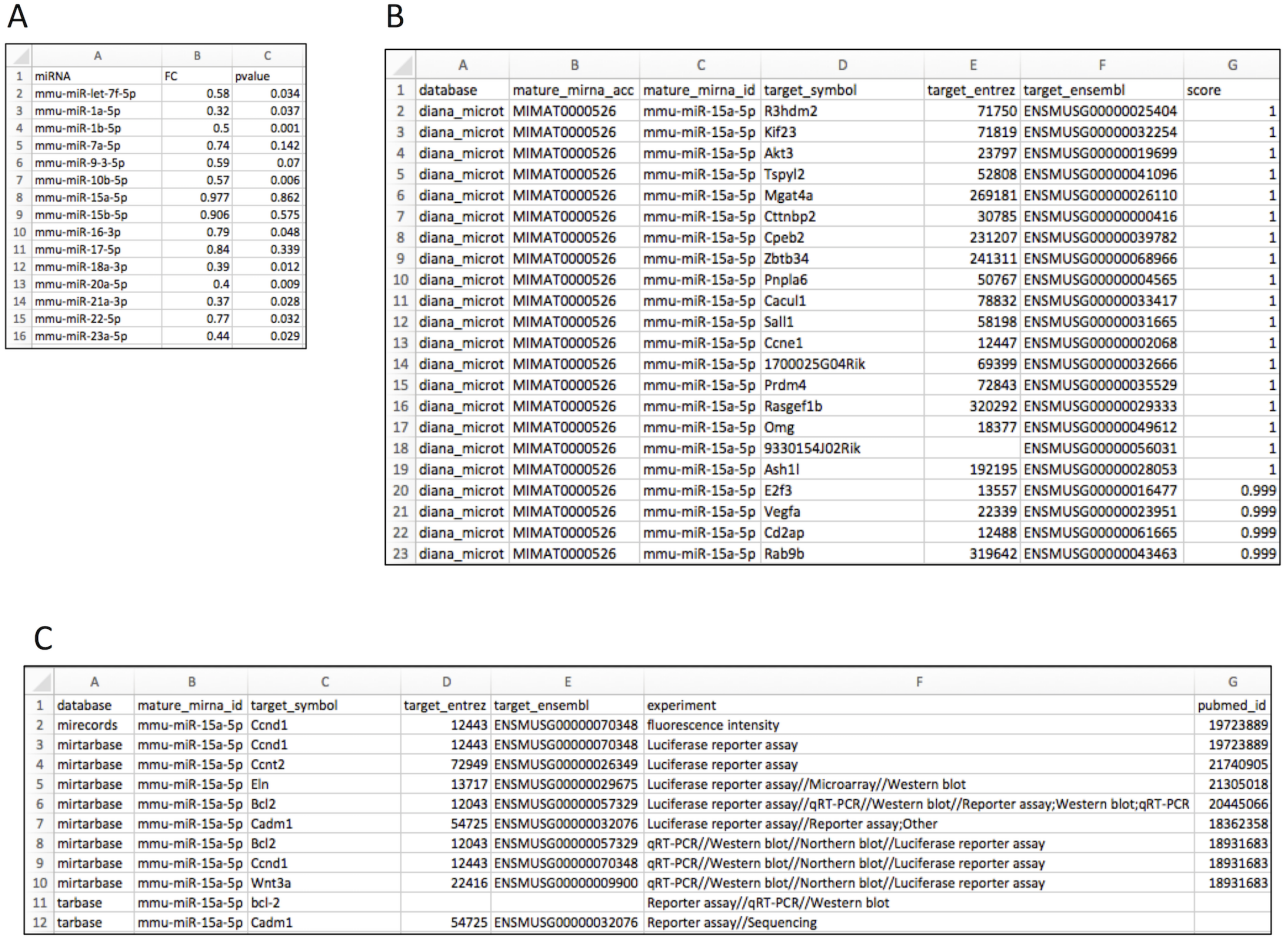
Layout for input and target mRNA mapping output files. (A) input file showing required column elements: miRNA name, fold change (FC) and *p* value; (B) output file layout showing results for the predicted target mRNA query; (C) output file layout showing results for the validated target mRNA query.

Figure 2A shows the input file layout. Upon importing the data to the R environment (sample data object name: *liver*), target mRNA mapping is conducted using the *rbiomirgs_mrnascan* function, through the command line: *rbiomirgs_mrnascan(objTitle = “mmu_liver_predicted”, mir = liver$miRNA, sp = “mmu”, queryType = “predicted”, addhsaEntrez = TRUE, parallelComputing = TRUE, clusterType = “FORK”)*. Figures 2B and 2C show truncated mapping results for both predicted and validated mapping results for miRNA *mmu-miR-25a-5p*. The mapping results showed that more predicted targets were retrieved than validated targets. The function output R projects as well as one *csv* file per miRNA tested. Since the case study enabled human ortholog Entrez ID conversion functionality, the function exported an R object including the Entrez ID for the human orthologs, with the suffix *“_hsa_entrez_list*” in the name.

Prior to enrichment, GS database files need to be obtained. For the case study, we used *gmt* files for KEGG and GO term databases downloaded from MSigDB (http://software.broadinstitute.org/gsea/msigdb). Regarding GO term, separated files were used for biological processes (BP) and molecular function (MF) databases. The case study used the predicted miRNA:mRNA interaction results for enrichment. Furthermore, since all GS database files were based on human genes, we used the human ortholog Entrez ID list. GS enrichment was carried out with the command line (using KEGG database as the example): *rbiomirgs_logistic(objTitle = “mirna_mrna_iwls”, mirna_DE = liver, var_mirnaName = “miRNA”, var_mirnaFC = “FC”, var_mirnaP = “pvalue”, mrnalist = mmu_liver_predicted_mrna_hsa_entrez_list, mrna_Weight = NULL, gs_file = ”kegg.v5.2.entrez.gmt”, optim_method = “IWLS”, p.adj = “fdr”, parallelComputing = TRUE, clusterType = “PSOCK”)*.

**Figure 3:**
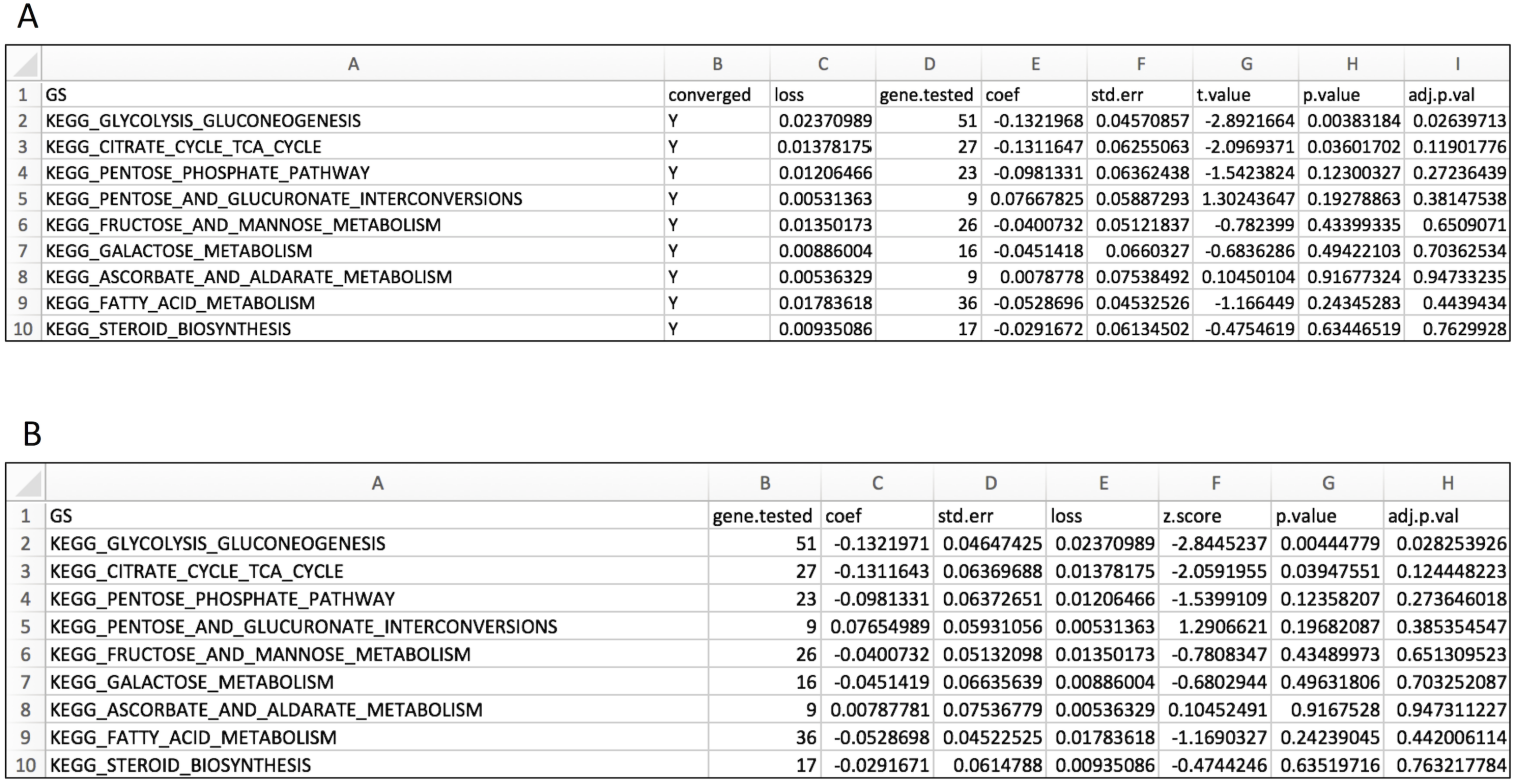
Layout for output GS enrichment results file for the case study, using two parameter optimization methods. (A) IWLS method; (B) BFGS method.

**Figure 4:**
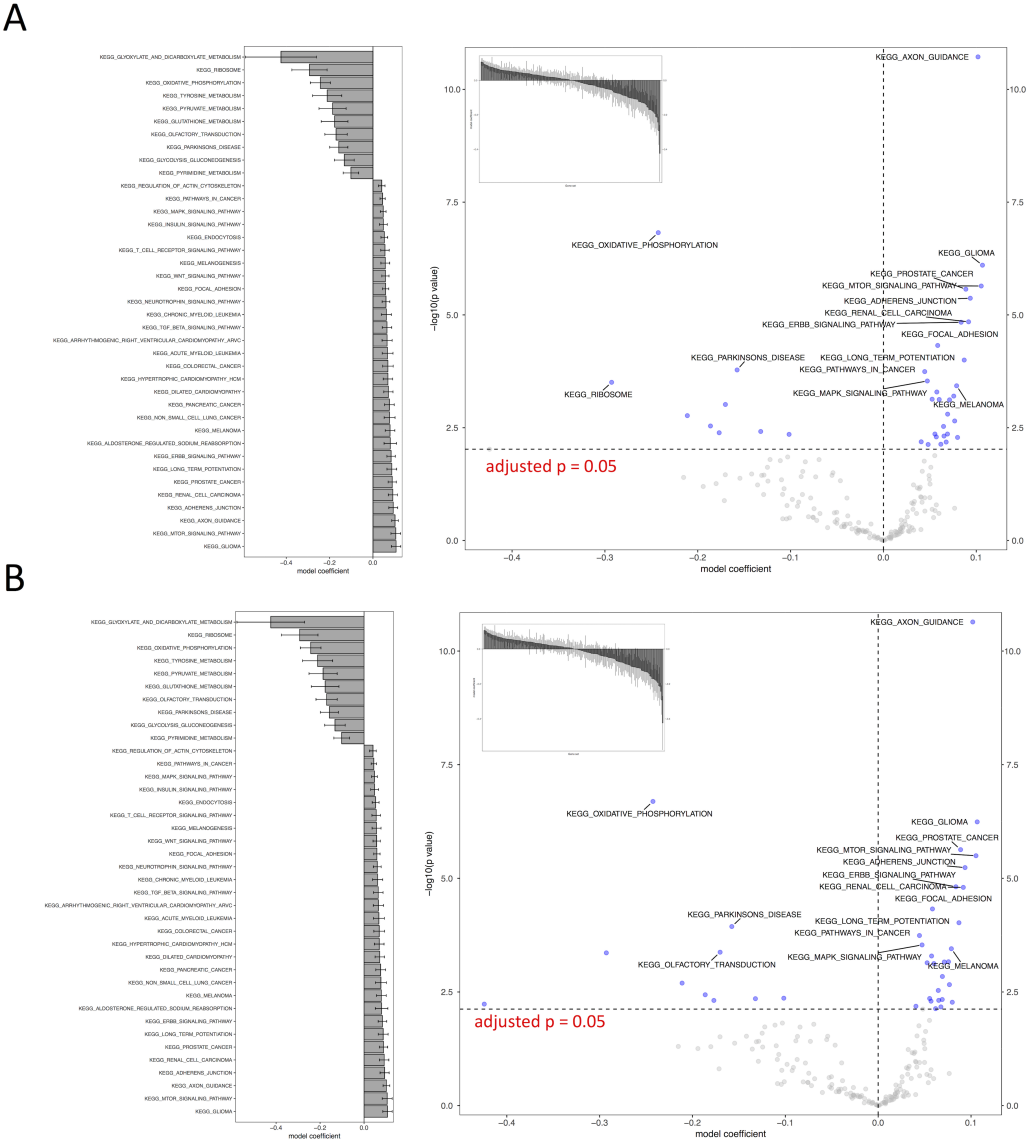
Visual representation of KEGG pathway analysis for the case study. Volcano plot depicts the significance and directionality distribution for the KEGG pathway tested (−log *p* value vs. model coefficient). Blue (upper quadrants) represent the significantly enriched KEGG pathways and the bar graph in the volcano plot shows the overall distribution of model coefficient. Top 15 most significantly enriched KEGG pathways are labeled. Bar graph shows the top 50 enriched gene sets; the bars are model coefficient ± standard error. Only the gene sets with an FDR adjusted *p* < 0.05 are plotted. (A) IWLS method; (B) BFGS method.

**Figure 5:**
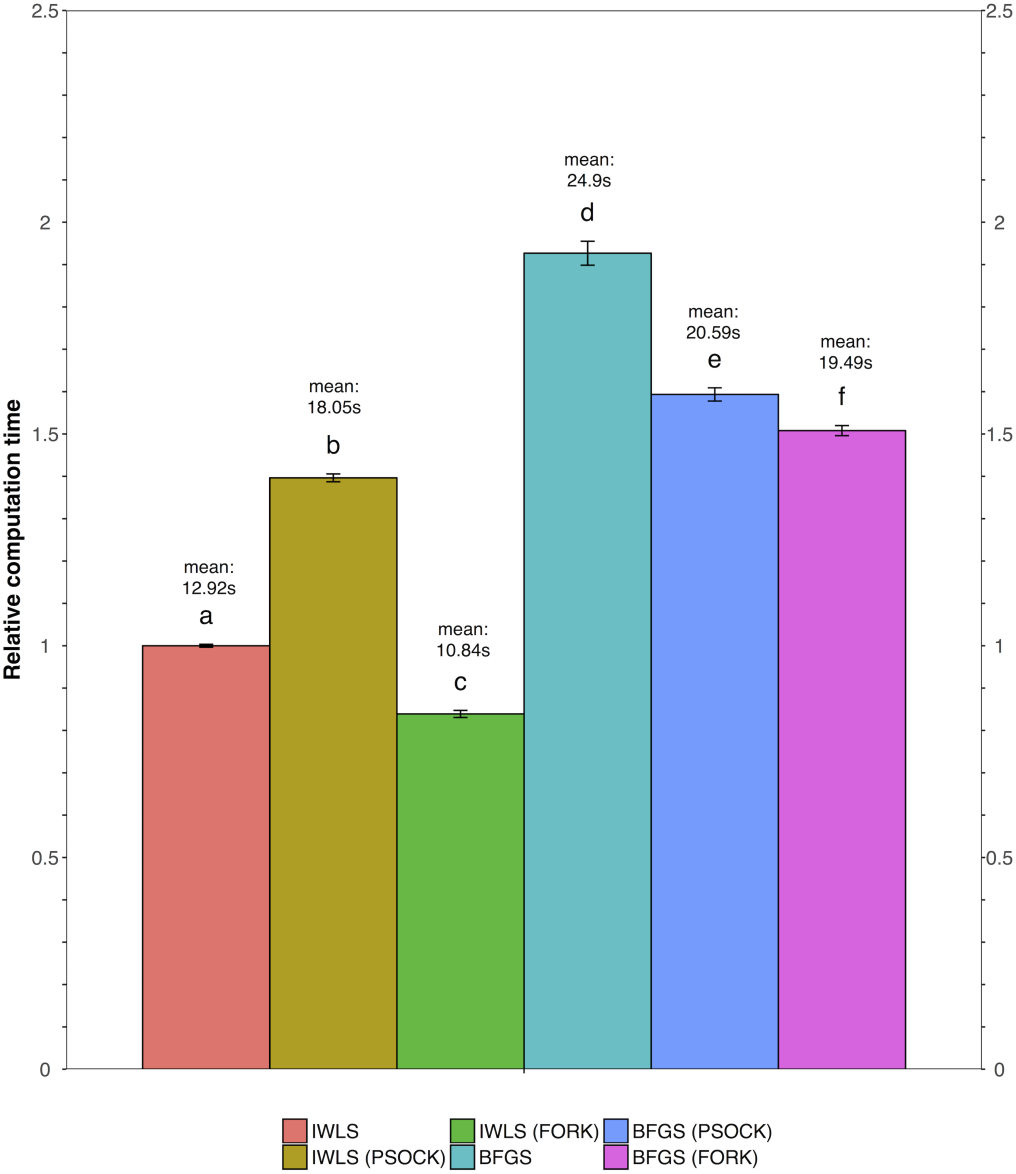
Comparison of relative computation time for parameter optimization between non-parallel and parallel settings using the KEGG database. FORK and PSOCK are parallel computing modes, with the former Unix or Unix-like operating system only. Bars are relative computation time ± SEM; different letters represent statistically significant changes (*p* < 0.05) according to a one-way ANOVA test with a Tukey post-hoc test. The raw mean value for each test is labeled in the graph based on three repeats.

We tested all three parameter optimization algorithms on the KEGG analysis to select for the most effective method. The KEGG database included 186 pathways. Firstly, the liver data failed to converge for all the gene sets tested using the L-BFGS-B algorithm. Figure 3 shows a truncated version of the IWLS and BFGS results. The results suggest that both methods led to consistent coefficient values and model significance (Figs. 3 and 4). We found that the IWLS method with parallel computing enabled with the Unix operating system exclusive FORK mode took the least amount of time to converge for KEGG analysis (Fig. 5, based on three repeats). The one-way analysis of variance (ANOVA) test on the computation time suggested the time reduction when using such configuration was significant (Fig. 5).

**Figure 6:**
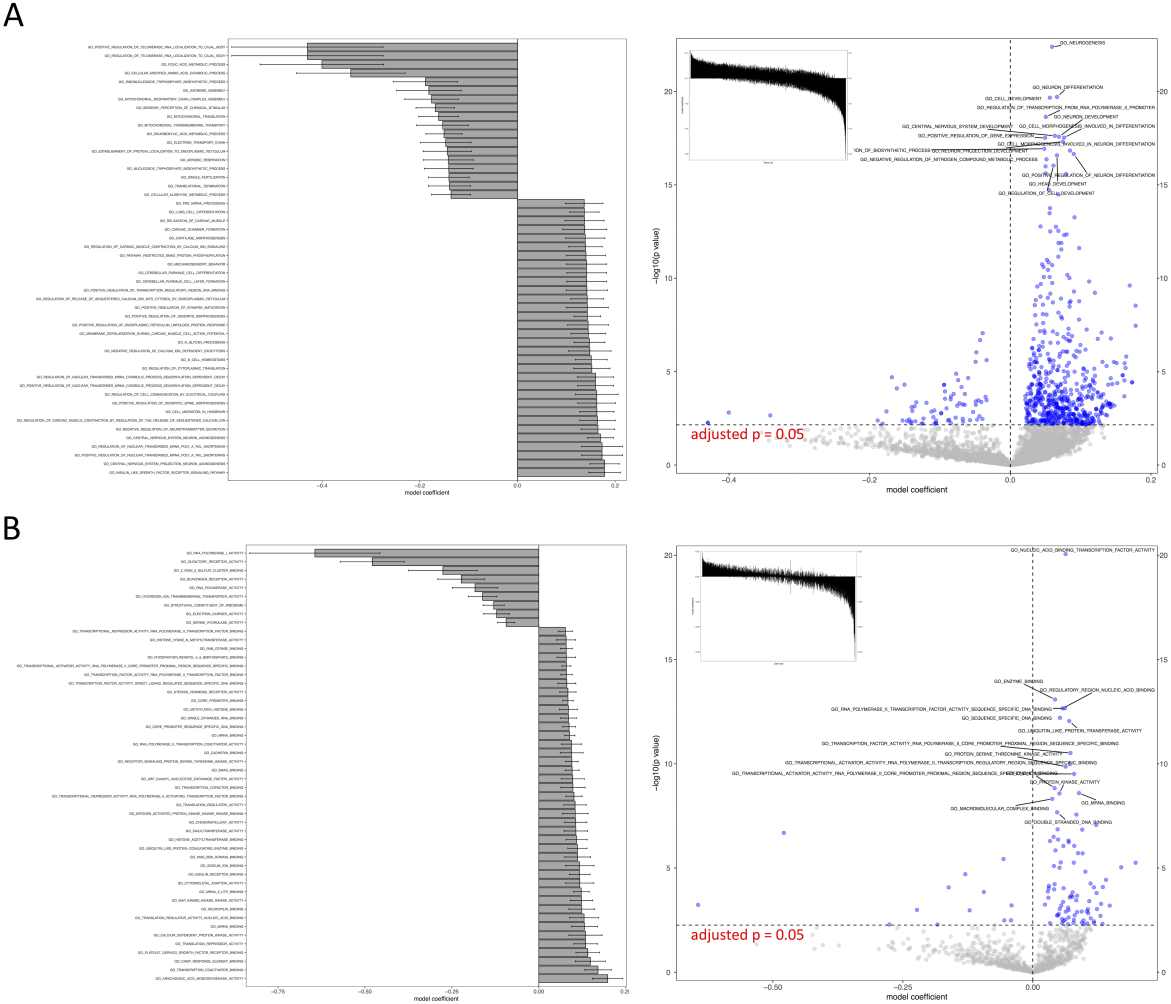
Visual representation of GO term analysis for the case study. (A) biological processes (BP); (B) molecular functions (MF). Additional information is same as Fig. 4.

As such, the following GO term enrichment was also carried out using IWLS and FORK methods. The results showed a similar trend as that of the KEGG analysis (Figs. 4 and 6), where more GO terms with a positive model coefficient value were identified.

## Discussion

RBiomirGS requires a miRNA identity list, a DE results list, as well as a GS database file as input (Fig. 1). The package uses fold change (FC) and *p* value to calculate the miRNA score, *S*_*mirna*_. Since the DE results are associated with the miRNAs, both miRNA identity and DE results can be provided in a single *csv* file. The data layout can be viewed in Fig. 2. In addition, due to the modularization of the package functionality, target mRNA mapping can be used as a standalone function, with a list of miRNA names as input. The GS database file can be downloaded from various sources. One such source is MSigDB, which indexes popular GS database such as KEGG and GO term. Naturally, databases from other sources can also be used.

To efficiently process high throughput datasets, RBiomirGS implements parallel computing across all major functions. Depending on the user’s computer configuration (i.e., number of CPU cores), parallel computing can provide significant speed enhancements. Moreover, both Unix/Unix-like operating system exclusive FORK and universal PSOCK modes are available for maximizing hardware compatibility. It is worth noting that this feature can be disabled by users. Function *rbiomirgs_logistic* also implements linear algebra for score calculation to reduce computation time.

The target mRNA mapping module also features an optional gene Entrez ID conversion functionality that searches for human gene orthologs on Ensembl databases for rodent models (i.e., mouse or rat). Given the high conservation level in miRNA primary structure across species, such function enables the potential of revealing the miRNA functional implication in human from rodent models. The human Entrez ID conversion function is built upon the open sourced Biomart platform (Durinck et al., 2005; Durinck et al., 2009). By integrating Biomart software into the package, RBiomirGS connects directly to Ensembl database (http://www.ensembl.org) for human ortholog search using the most up-to-date information. While beneficial, such configuration imposes one limitation of the package wherein an active and functional internet connection is required for the target mRNA mapping function.

RBiomirGS conducts GS analysis through mRNA scores, miRNA scores and logistic regression. The mRNA score *S*_*mrna*_ is based on the assumption that, in most cases, miRNAs inhibit target mRNA translation events. Therefore, *S*_*mrna*_ represents the inhibitory effect on the mRNA of interest. As the sign reversed summation of *S*_*mirna*_, the biological interpretation of *S*_*mrna*_ can be described as the following: In the case of a two-group comparison (i.e., experimental vs control), a positive *S*_*mrna*_ means the mRNA of interest might be inhibited more in the control group, whereas a negative value means the mRNA might be under miRNA inhibition upon experimental conditions. In addition, a bigger absolute value represents a stronger miRNA inhibitory effect. Given that *S*_*mirna*_ contains directionality information, such approach allows for accumulation and cancelation effects on the mRNA when the mRNA of interest is targeted by multiple miRNAs. Since the strength of the interaction between miRNA and mRNA varies among different miRNAs, it is critical to incorporate such consideration into the *S*_*mrna*_ calculation, regardless of the availability of such measurement. Therefore, we added the weight term *w* to Eq. (2) to accommodate the affinity of the miRNA:mRNA interaction, should such metric be available.

The central goal of the current logistic regression-based classification modelling is to separate the members of a gene set from the rest of the genes using *S*_*mrna*_, which represents the overall miRNA regulatory effect. If a gene can be categorized into a gene set solely based on its *S*_*mrna*_, then said gene set is under miRNA-dependent regulation. As such, based on the model significance test and user customizable GS *p* value threshold (e.g., FDR adjusted *p* value < 0.05 by default), a GS model with a significant adjusted *p* value means that the membership to such gene set for a gene can be determined based on its *S*_*mrna*_, or that the gene set is significantly impacted by miRNA regulation. The biological interpretation of the model coefficient from Eq. (4) can be stated as follows (again, in the context of two-group comparison, i.e., experimental vs control): if the coefficient is positive, miRNA inhibition on target mRNAs might be lifted, thereby leading to less suppression on the gene set of interest in the experimental group. Furthermore, with a positive coefficient, a unit increase in *S*_*mrna*_ results in an increased odds ratio of a gene belonging to the gene set of interest. Conversely, a negative value means the opposite. It needs to be clarified that a positive model coefficient for a gene set means that the gene set of interest might be under more miRNA-dependent inhibition in the control group, as opposed to being activated under the experimental condition. Such observation is closely related to the fact that the miRNA regulation on a pathway is mostly indirect, and represents only one layer of regulation on the mRNAs. As such, another limitation of RBiomirGS is in its limited capacity for evaluating gene set activation when solely relying on miRNA DE results.

The case study demonstrated the usage of RBiomirGS. In general, enrichment on all three GS databases suggested that more gene sets were free from miRNA-dependent inhibition in the livers of torpid marsupials, represented by positive model coefficient values (Figs. 4 and 6). The result is consistent with the observation from the original study where most miRNAs tested showed decreased relative expression levels in liver (Hadj-Moussa et al., 2016), leading to less inhibitory effect on their target mRNAs, which in turn resulted in more gene sets independent from miRNA-dependent regulation. For example, such enriched KEGG pathways included mTOR signaling pathway and MAPK signaling pathway, which, when activated, were considered to play critical roles in facilitating torpor (Hadj-Moussa et al., 2016). However, the volcano plots in Figs. 4 and 6 suggest that potentially inhibited gene sets in the liver from torpid marsupials exhibited a greater impact by the miRNA, i.e., a wider spread pattern on the *x*-axis in the negative direction. The KEGG pathways that might be suppressed included Ribosome (KEGG ID: map03010), RNA polymerase (KEGG ID: map03020), Oxidative phosphorylation (KEGG ID: map00190), and Pyruvate metabolism (map00620). Inhibition of those pathways may contribute to suppressing ATP expensive cellular processes such as global gene transcription and protein synthesis, all of which have been reported to be inhibited in other hibernating animals (Storey, 2010; Wu & Storey, 2016). It is also not a surprise that oxidative phosphorylation and pyruvate metabolism pathways were inhibited under hypometabolic conditions (Storey, 1997). Overall, by using RBiomirGS, additional miRNA-dependent regulatory mechanisms that underpin the molecular adaptations facilitating daily torpor in marsupials were revealed.

By incorporating all the core steps into one R package, RBiomirGS eliminates the need for switching between different software packages, or between different software platforms. The package also provides two data visualization functions that can produce three types of plots. Furthermore, the modular structure of RBiomirGS enables various access points to the analysis, with which users can choose the most relevant functionalities for their workflow. With RBiomirGS, users will be able to comprehensively assess the functional implications of the miRNA expression profile under the corresponding experimental condition by minimal input and intervention. Accordingly, RBiomirGS provides an all-in-one and highly accessible miRNA GS analysis solution.

##  Supplemental Information

10.7717/peerj.4262/supp-1 Supplemental Information 1 Supplemental data for the sample study This file contains target mRNA search results, and gene set analysis results.

## Additional Information and Declarations

### Data Availability

The following information was supplied regarding data availability: GitHub: http://github.com/jzhangc/RBiomirGS.
